# Estimation of the Use of Antibiotics in the Small Ruminant Industry in the Netherlands in 2011 and 2012

**DOI:** 10.1371/journal.pone.0105052

**Published:** 2014-08-12

**Authors:** Inge Santman-Berends, Saskia Luttikholt, René Van den Brom, Gerdien Van Schaik, Maaike Gonggrijp, Han Hage, Piet Vellema

**Affiliations:** 1 Department of epidemiology, GD Animal Health, Deventer, The Netherlands; 2 Department of small ruminant health, GD Animal Health, Deventer, The Netherlands; 3 Department of cattle health, GD Animal Health, Deventer, The Netherlands; The University of Melbourne, Australia

## Abstract

The aim of this study was to estimate the quantity of antibiotics and classes of antibiotics used in the small ruminant industry in the Netherlands in 2011 and 2012. Twelve large veterinary practices, located throughout the Netherlands were selected for this study. All small ruminant farms associated with these practices that had complete records on the quantity of antibiotics prescribed were included. The veterinary practices provided data on all antibiotics prescribed, and the estimated animal used daily dose of antibiotics per year (AUDD/Y) was calculated for each farm. The median AUDD/Y in small ruminant farms was zero in both years (mean 0.60 in 2011, and 0.62 in 2012). The largest quantity of antibiotic use was observed in the professional goat industry (herds of ≥32 goats) with a median AUDD/Y of 1.22 in 2011 and 0.73 in 2012. In the professional sheep industry (flocks of ≥32 sheep), the median AUDD/Y was 0 in 2011 and 0.10 in 2012. In the small scale industry (flocks or herds of <32 sheep or goats), the median AUDD/Y never exceeded 0. The most frequently prescribed antibiotics in the small scale industry and professional sheep farms belonged to the penicillin class. In professional goat farms, antibiotics of the aminoglycoside class were most frequently prescribed. This study provides the first assessment on the quantity of antibiotic use in the small ruminant industry. Given a comparable attitude towards antibiotic use, these results might be valid for small ruminant populations in other north-western European countries as well. The antibiotic use in the small ruminant industry appeared to be low, and is expected to play a minor role in the development of antibiotic resistance. Nevertheless, several major zoonotic bacterial pathogens are associated with the small ruminant industry, and it remains important that antibiotics are used in a prudent way.

## Introduction

In the Netherlands, sheep and goats are generally kept as companion animals. However, there is also a substantial number of professional farms with small ruminants that produce milk and meat for human consumption. In the latter, food safety and food quality are of great importance. To ensure a production of meat and milk by healthy livestock, sick animals need to be treated in a responsible manner. However, the use of antibiotics and other medicines may enhance the development of antimicrobial resistance (AMR) [1]–[3]. Additionally, the fact that the same classes of antibiotics are used in veterinary and human medicine is a reason for concern [4]. In the last decade, antibiotic resistance in livestock has become a great concern in many European countries because of the association between livestock and the presence of resistant bacteria [5], [6]. Nevertheless, the role of small ruminants in this discussion might be of minor importance because earlier research did only find low rates of AMR in sheep [7].

In December 2008, the Dutch Ministry of Agriculture agreed to a covenant entitled “Antibiotic resistance in livestock” for the pig, poultry, cattle and veal industry [8]. The goal of this covenant was to monitor and reduce the use of antibiotics, and therewith achieve a decline in antibiotic resistance in these livestock industries. This resulted in a reduction of the use of antibiotics of over 50% in 2012 relative to the use in 2009 [9]. Because the small ruminant industry did not participate in this covenant, the use of antibiotics in this industry has not been monitored.

From January 1^st^ 2010 onwards, everyone who owns small ruminants in the Netherlands is obliged to register their sheep or goats in the Identification and Registration (I&R) database. The quality of this national database has improved over time, and appears to provide a reasonable and relatively complete representation of the Dutch small ruminant industry in both 2011 and 2012. The combination of improved registration in the central I&R-database and improved registration of prescribed antibiotics in the databases of veterinary practices offered the opportunity to estimate the quantity of antibiotics that were used in the small ruminant industry. Beforehand, the general impression was that the antibiotic use in this industry is fairly low, especially compared to other livestock industries. However, there was no information available on the amount and type of antibiotics used in the small ruminant population. The aim of this study therefore was to estimate the quantity and types of antibiotics that were used in the small ruminant industry in the Netherlands in 2011 and 2012.

## Materials and Methods

### Ethics Statement

The data that were used for this study belonged to the veterinary practices involved. They gave consent to use the data for this study, given that all data of small ruminant holders and the veterinary practice were anonymised prior to analysis. After combining the different datasets, all identifying information such as names, addresses and unique herd identification (UHI) numbers from both the small ruminant holders and their veterinary practices were either removed or were anonymised. This was done prior to analysis and in this way, it was impossible to trace the data and results to either small ruminant holders or veterinary practices. The Dutch government and the farmers organisation were informed and agreed to this procedure prior to this study.

### Study population

For this study, veterinary practices with a minimum of fifty small ruminant holders as client were asked to participate. Eventually twelve large veterinary practices that were located throughout the country were included. The study population consisted of 5,399 holders of small ruminants that were clients of these veterinary practices. The Netherlands had a total of 34,806 registered small ruminant farms in 2012 [10], thus 16% of all registered holdings with small ruminants were covered. Based on species (sheep/goat) and herd size (≤ or > than 32 heads), herds were divided into four different subtypes of small ruminant farms: small scale sheep farms, professional sheep farms, small scale goat farms and professional goat farms. The last group contained both dairy and non-dairy farms. The cut-off value of 32 head was consistent with earlier studies in the Netherlands [11] and was agreed upon by the different stakeholders in the Dutch small ruminant industry.

### Available data

The twelve veterinary practices provided data on each delivery of medicines or services to their small ruminant holders. Because four different management systems were used in the veterinary practices that were included in this study, data from the different systems were assigned appropriately before they were combined.

The data from the veterinary practices consisted of names and addresses of clients, services or medicines involved, the species for whom the medicines were provided, date or year of delivery, and delivered amount of prescribed medicines. To be able to calculate quantity of antibiotic delivered to an average animal in the small ruminant industry, data from the veterinary practices were combined with three other data sources, namely:

1. **Animal demographic information** (GD Animal Health) and Ministry of Economic Affairs (EZ)) information on herd level: unique herd identification numbers (UHI), names and addresses of the farms, livestock species present on the farm (sheep/goats/cattle/pigs/poultry). On animal level, this data contained species, birth dates, dates of arrival and removal, reason for arrival (birth, purchase, import), and reason for removal (sale, export, slaughter, death).
2. **Weight information** (Agricultural Economical Institute (LEI)) containing standardized weights for sheep and goats, divided into two and three different age categories respectively.
3. **Pharmacological information** (Faculty of Veterinary Medicine, Utrecht University) containing all relevant information from the manuals of the antibiotics that were approved for use in (small) ruminants. This data included names and registration numbers of products that contain antibiotics, type and concentrate of active substance, and the weight of sheep or goat that could be treated with a certain amount (often mL or g) of active substance.

To be able to combine the different datasets, prior to the start of the validation, the data from the veterinary practices were combined with the demographic information to obtain the UHI numbers for each herd. This was in consent with all stakeholders involved (see also the ethics section). Names and addresses were removed from the data and the UHI numbers were anonymised before the data were analysed.

### Data validation

The data from the veterinary practices contained 7,483 records with prescribed antibiotics to be used for small ruminants present on the farms in the years 2011 and 2012.

**Figure 1:**
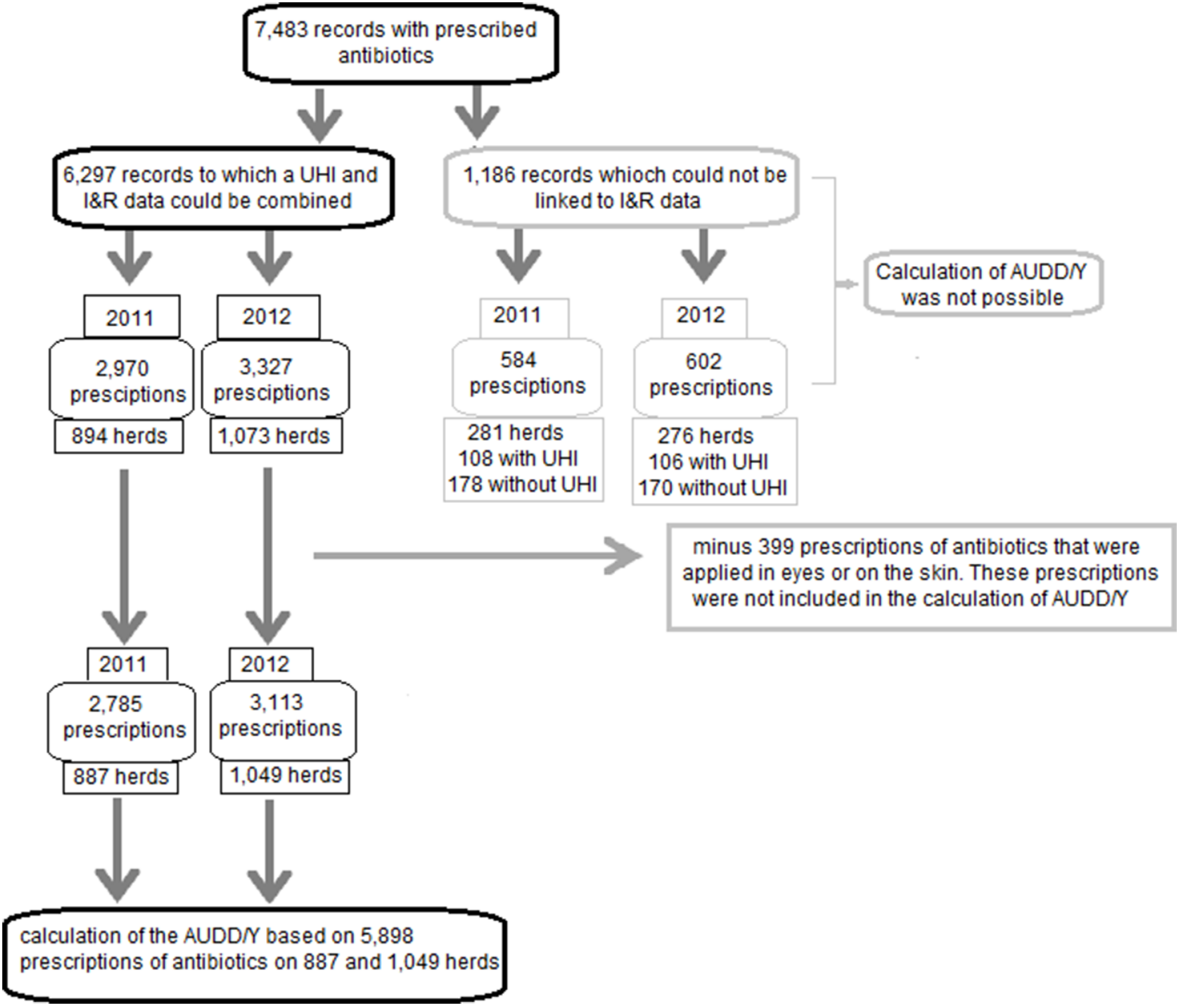
A schematic overview of the validation process of the data of prescribed antibiotics that were provided by the veterinarians for the calculation of the estimated animal used daily dose of antibiotics used per year (AUDD/Y) in farms with small ruminants in 2011 and 2012.

These data were combined with animal demographics, weight and the pharmacological information in order to calculate the amount of active substances that were delivered to the herds and the total weight of the small ruminants treated. The data in the I&R database were incomplete: for several farms data about the number of animals present was missing. Therefore only 6,297 out of 7,483 antibiotic supply records (84%) were available to calculate the Animal Used Daily Dose of antibiotics per year (AUDD/Y) (Figure 1). The calculated AUDD/Y does not present the truly used daily dose, but presents an estimation of this parameter because 1) the available data contained the amount of antibiotics delivered which may not be completely used by the farmer and 2) for the calculations average weights are used instead of the exact weights of all small ruminants in the herds.

Of those 6,297 prescriptions of antibiotics, 2,970 were delivered to 894 different farms in 2011, and 3,327 were delivered to 1,073 different farms in 2012. Of the remaining 1,186 prescriptions, 584 were delivered to 281 different farms in 2011, and 602 prescriptions were delivered to 276 different farms in 2012. Of 103 and 106 of these herds respectively, a UHI was available, but not registered in the I&R database. Of the remaining 178 and 170 farms respectively, only names and addresses were available that could not be combined with an UHI number to identify individual farms.

The weight (in kilograms) of animals present on the farm was one of the requirements to be able to calculate the AUDD/Y. Since this information was unknown for 281 and 276 farms as described above, it was not possible to include these herds in the calculations of the AUDD/Y. Nevertheless these prescriptions of antibiotics were included for descriptive purposes.

For some antibiotics the prescribed dosage differs between sheep and goats, and in a few cases it was not specified for which species the antibiotics were used. In those cases we decided to include the highest prescribed dose in our calculations.

Finally, farmers use different amounts of ointment to treat eye and skin problems. Therefore it was not possible to determine the animal weight in kilograms that was treated per tube of ointment, resulting in an inaccurate estimation of the AUDD/Y for these applications. For this reason we decided to remove those prescribed antibiotics that are applied locally e.g. ointment in eyes or on the skin. This was in consensus with the calculations of the AUDD/Y in other animal species in the Netherlands [12].

Eventually, 2,785 and 3,113 prescriptions of antibiotics, in 887 and 1,049 farms in 2011 and 2012 respectively, remained for the calculation of the AUDD/Y in small ruminant herds that received antibiotics from their veterinary practice (Figure 1).

**Figure 2:**
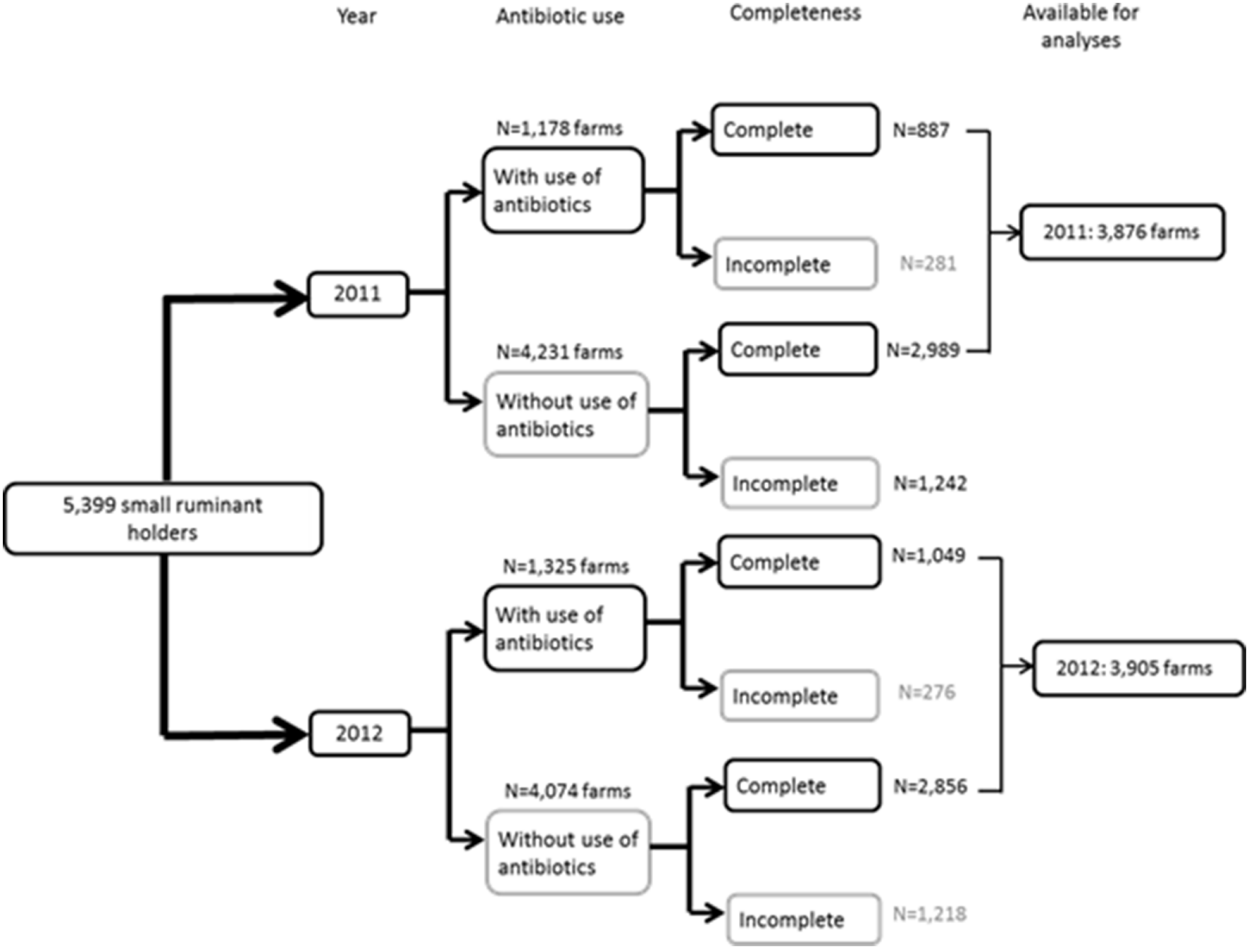
A schematic overview of the number of farms with small ruminants that were clients of the twelve veterinary practices in this study. The figure shows the number of farms with and without antibiotic use and with complete and incomplete data (missing information on the number of animals present in the herd) for both 2011 and 2012.

Of the 4,231 and 4,074 farms to which veterinary practices had not prescribed antibiotics in 2011 and 2012 respectively, only 2,989 (71%) and 2,856 (70%) farms had complete I&R data (Figure 2). In accordance with the selection criteria to include only farms with complete data, and analogous to the farms to which antibiotics were prescribed during the analysed period, we decided to include only farms with complete data (2,989 and 2,856 herds in 2011 and 2012, respectively). Lastly, for the calculation of the AUDD/Y in the entire small ruminant industry, data from 3,876 and 3,905 farms with small ruminants respectively, were used for the analyses (Figure 2).

### Analyses

All analyses were carried out with STATA version 13 [13]. For each of the 887 and 1,049 farms that had been prescribed antibiotics for their small ruminants in 2011 and 2012 respectively, the total animal weight treated was calculated for each delivery. Weight was used instead of number of animals because antibiotics are prescribed by weight. The total animal treated weight per supply of antibiotics was calculated by multiplying the amount of delivered product (product_i_) by the content of active substance (active substance_i_) according to the manual of the product. The total amount of active substance divided by the prescribed dose per kg animal (prescribed dose_i_ per kg) resulted in the total treated animal weight of the small ruminants per herd in kilogram (kg treated) (formula 1).
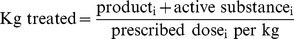
(1)Where:

Kg treated : the total weight of sheep/goats in kilograms treated with the antibiotics prescribed by the veterinarian per treatment
Product_i_ : the total amount of prescribed antibiotic per unit i
Active substance_i_ : the content of active substance per unit i of the antibiotic
Prescribed dose_i_ per kg : the total weight of small ruminant in kilograms treated with a predefined concentration (unit i) of active substance
i : mg/gram/mL/piece/pastille etc.

Subsequently, the animal used daily dose of antibiotics (AUD) per prescription was calculated by dividing the total animal weight that was estimated to be treated with antibiotics by the total amount of weight of the sheep/goats in kilograms (kg small ruminant) present in the herd.

Of the 5,989 prescriptions of antibiotics, the exact date of delivery was known for 4,803 deliveries. Of the remaining 1,095 prescriptions only the year of delivery could be provided by the veterinary practices. If the exact date of delivery was known the amount of treated weight was divided by the total amount of weight in kilograms present on that date, otherwise the average weight in the year of delivery was used.

The weight of the small ruminants in the herd was based on the number of sheep and/or goats in each age category, multiplied by the corresponding standard weight. The standard weights differentiate sheep and goats into five categories (Table 1).

**Table 1:** Description of the different weight and age categories that were used by the Agricultural Economical Institute (LEI) to differentiate between species and ages within the small ruminant industry.

Species	Category	Weight in kg	Age in days
Sheep	Ewe	75	>365
Sheep	Lamb	22	0–365
Goat	Milking goat	75	>365
Goat	Rearing kid	37,5	31–365
Goat	Kid	7	0–30

The animal used daily dose (AUDD) per prescription was summed to calculate the AUDD/Y for each herd in 2011 and 2012 (Formula 2).
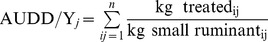
(2)Where:

AUDD/Y : estimated animal used daily dose of antibiotics per year
Kg treated : the total weight of sheep/goat in kilograms treated with the antibiotics prescribed by the veterinarian
Kg small ruminant : the total weight of small ruminant in kilograms present in the herd at the moment the antibiotics are prescribed
i : each of the antibiotic deliveries
j : 2011 or 2012

Herds that did not receive any antibiotics in a year had an AUDD/Y of 0. Descriptive statistics were used to describe the AUDD/Y for the entire small ruminant industry and for each small ruminant subtype. Median tests [14] and proportion tests were used to evaluate differences in proportion of herds with antibiotic use, and to evaluate differences in the AUDD/Y between small ruminant subtypes.

The data that were used for this study are freely available upon request according to the data sharing policies of PLOS ONE. Requests can be directed to the small ruminant department of GD Animal Health in the Netherlands.

## Results

### Descriptives

The twelve veterinary practices had an average of 423 clients with small ruminants, of which a median of 84 (mean 99) and 120 (mean 113) received antibiotics in 2011 and 2012 respectively. The number of farms for which each veterinary practice prescribed antibiotics varied from 20 to 246 in 2011, and from 26 to 232 in 2012 (Table 2).

**Table 2:** Descriptive results of the number of connected small ruminant farmers, the number of farmers that were prescribed antibiotics, and the number of times antibiotics were prescribed by the twelve veterinary practices that cooperated in this study in the Netherlands.

Year	Median [mean] number of farms per veterinary practice	Number of farms for which antibiotics were prescribed		Number of times antibiotics were prescribed by the veterinary practices	
		Median [mean]	Range	Median [mean]	Range
2011	423 [415]	84 [99]	20–246	261 [296]	77–841
2012	423 [415]	120 [113]	26–232	299 [327]	74–779

In total, the twelve practices prescribed antibiotics to small ruminant holdings 7,483 times during the analysed period. The median number of times each veterinary practice prescribed antibiotics to herds with small ruminants was 261 in 2011, and 299 in 2012. The number of prescriptions of antibiotics ranged from 77 to 841 in 2011, and from 74 to 779 in 2012 (Table 2).

All small ruminant farms with complete data were classified into one of the four small ruminant subtypes (Table 3).

**Table 3:** The number of farms included the study with or without the use of antibiotics in 2011 and/or 2012 for each subtype of small ruminant farm in the Netherlands.

Small ruminant farm subtype		With antibiotic use		Without antibiotic use		% with antibiotic use
		dairy	other	dairy	other	
Professional goat farms (≥32 goats)	2011	46	15	0	11	85
	2012	51	18	0	11	86
Professional sheep farms (≥32 sheep)	2011	0	566	0	636	47
	2012	0	686	0	502	58
Small scale goat farms (<32 goats)	2011	0	52	0	566	8
	2012	0	51	0	582	8
Small scale sheep farms (<32 sheep)	2011	0	208	0	1,776	10
	2012	0	243	0	1,761	12

Within the professional goat farms subtype that received antibiotics, 75% and 74% of the farms (46 in 2011, and 51 in 2012) were dairy goat farms. All of the dairy goat farms received antibiotics at least once in both years. The non-dairy professional goat farms were often much smaller than the dairy goat farms and also received antibiotics less often (15 out of 26, and 18 out of 29 non-dairy professional goat farms received antibiotics in 2011 and 2012 respectively). The percentage of farms for which antibiotics had been prescribed ranged from 8% in the small scale goat farms to 85% and 86% in the professional goat farms (Table 3).

The dairy goat farms were the largest herds with a median herd size of 834 and 833 goats in 2011 and 2012, respectively. These herds were part of the professional goat farms subtype, which had a median herd size of 746 and 691 goats in 2011 and 2012. With these herd sizes, the professional goat farms that used antibiotics and were included in this study were somewhat larger compared to the average professional goat herds in the Netherlands (median 658 in 2012). In addition, small scale goat farms that used antibiotics in 2011 and/or 2012 were larger as well, compared to the average small scale goat farm in the Netherlands (median 11 vs. median 3 in both years).

In the professional and small scale sheep farms that used antibiotics in 2011 and/or 2012, there was a median of 64 and 13 sheep in 2011, and 70 and 13 sheep in 2012 respectively. These herd sizes were comparable to the median herd sizes of all professional and small scale sheep farms in the Netherlands (median 65 and 11 in 2012).

**Figure 3:**
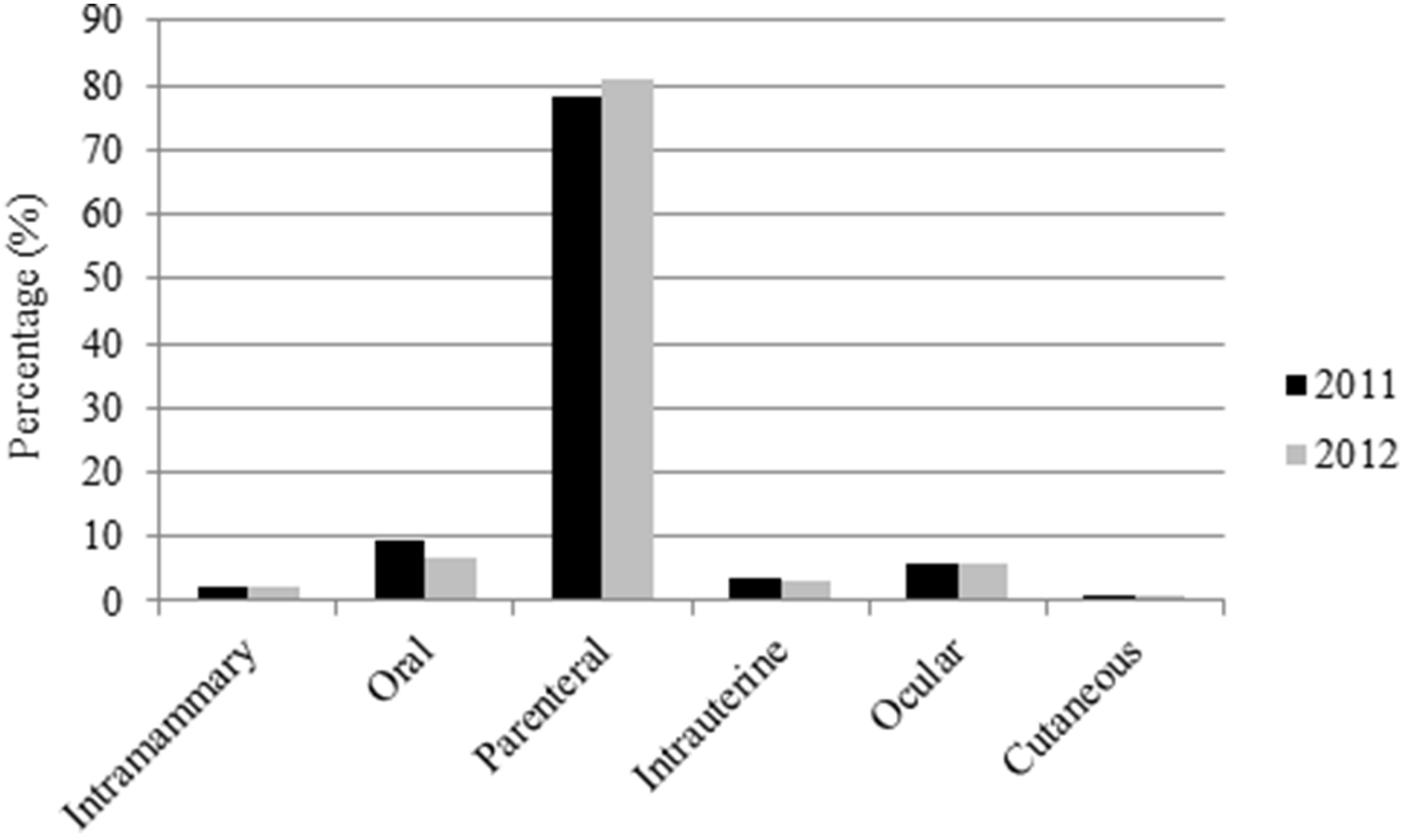
The application methods of antibiotics for small ruminants in 2011 and 2012 in the Netherlands.

Most antibiotics prescribed to small ruminants in this study were applied parentally (78% in 2011 and 81% in 2012) or orally (10% and 7%, respectively). In sporadic cases antibiotics were applied by intramammary or intrauterine routes (Figure 3). The third most used method of application of antibiotics in small ruminants was by means of ointments on the eyes, however, antibiotics that were applied as ointments on the eyes or skin (cutaneous) were excluded for the calculation of the AUDD/Y. (See 2.3 Data validation).

### Animal used daily dose of antibiotics per year

**Figure 4:**
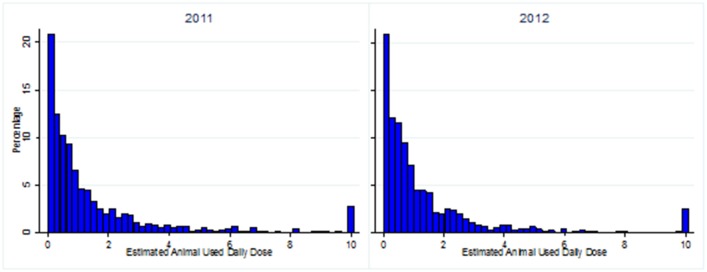
The distribution of the estimated Animal Used Daily Dose of antibiotics per year (AUDD/Y) in farms with small ruminants for which antibiotics were prescribed in 2011 and 2012. Values for AUDD/Y above ten were a rare event and are therefore set at ten for clarity of the figure.

For the calculation of AUDD/Y data on antibiotic deliveries and average standard weighs were used. Therefore, the AUDD/Y in this study presents an estimation of the AUDD/Y. The median AUDD/Y in herds with small ruminants that used antibiotics was 0.73 and 0.70 (mean 2.73 and 2.26) in 2011 and 2012 respectively (Table 4). However, there was a large variation in the AUDD/Y between herds, with many farms having an AUDD/Y slightly above 0, and only a small number of farms with a high AUDD/Y (Figure 4). The values of AUDD/Y in farms with small ruminants ranged from 3.7×10^−4^ to 181 in 2011, and from 1.8×10^−5^ to 219 in 2012 (Table 4). The AUDD/Y in the years 2011 and 2012 in farms with antibiotic use were not significantly different from each other. When farms without antibiotics use in one or both years were included as well, the median AUDD/Y was 0 (mean 0.62 in 2011, and 0.60 in 2012).

**Table 4:** The percentage of farms for which antibiotics were prescribed, and the estimated animal used daily dose of antibiotics per year (AUDD/Y) in 2011 and 2012 in all farms with small ruminants in the Netherlands, and in farms with antibiotic use only.

	% farms for whichantibiotics were prescribed	Median [mean] AUDD/Y in allsmall ruminant farms	Median [mean] AUDD/Y infarms with antibiotic use	Range in AUDD/Y on farms withantibiotic use
2011	23%	0 [0.62]	0.73 [2,73]	3.7×10^−4^–181
2012	27%	0 [0.60]	0.70 [2,26]	1.8×10^−5^–219

In 25 and 27 farms (3%) with small ruminants that used antibiotics in 2011 and 2012 respectively, an AUDD/Y above ten was found. Of the 25 farms with an AUDD/Y above ten in 2011, 18 farms only housed small ruminants, 6 farms also housed cattle, and one farm housed multiple other livestock species besides small ruminants. Of the 27 farms with an AUDD/Y above ten in 2012, 15 only housed small ruminants, ten farms also housed cattle, and two farms housed multiple other livestock species. Out of the seven and eight herds in 2011 and 2012 with the highest AUDD/Y (≥50), three and four farms (50%) had a combination of small ruminants and cattle on their farm. This percentage was higher than the percentage of combined farms with small ruminants and cattle in the whole studied population (34%), but this difference was not significant (*P*-value>0.05).

As stated previously, the percentage of subtypes of farms with small ruminants that used antibiotics ranged from 8% to 85% in 2011 and from 8% to 86% in 2012. Corrected for the percentage of farms with antibiotic use, the median AUDD/Y varied from 0 in both types of small scale holders, and professional sheep farms, to 1.22 in professional goat farms in 2011. In 2012, the median AUDD/Y in all farms that were included ranged from 0 in both types of small scale holders, and 0.10 in professional sheep farms, to 0.73 in professional goat farms (Table 5). Professional sheep and goat farms had a significantly higher AUDD/Y compared to the small scale sheep and goat farmers (*P*-Chisq<0.001). In addition, professional goat farms also had a significantly higher AUDD/Y than the professional sheep farms in both years (*P*-Chisq<0.001).

**Table 5:** The percentage of farms in this study for which antibiotics were prescribed, and the estimated animal used daily dose of antibiotics per year (AUDD/Y) in 2011 and 2012 for farms with antibiotic use and all farms with small ruminants in the Netherlands, per subtype of small ruminant farms.

		% farms for which antibioticswere prescribed	Median [mean] AUDD/Y onfarms with antibiotic use	Median [mean] AUDD/Y on all small ruminant farms
Professional goatfarms (≥32 goats)	2011	85%	1.57 [16.84]	1.22 [14.27]
	2012	86%	1.27 [8.00]	0.73 [6.81]
Professional sheepfarms (≥32 sheep)	2011	47%	0.60 [0.96]	0 [0.45]
	2012	58%	0.59 [1.10]	0.10 [0.63]
Small scale goatfarms (<32 goats)	2011	8%	1.52 [2.13]	0 [0.18]
	2012	8%	1.47 [5.48]	0 [0.44]
Small scale sheepfarms (<32 sheep)	2011	10%	1.61 [3.55]	0 [0.37]
	2012	12%	1.19 [3.20]	0 [0.39]

Most antibiotics were prescribed in the first months of the year, with the highest quantity in March. In the Netherlands, these months represent the lambing season [15]. Especially in dairy herds, almost no antibiotics were prescribed in the period when no lambs were born, i.e. between September and January. In the non-dairy herds, besides the prescriptions in the lambing season, antibiotics were prescribed at a low rate throughout the year.

**Figure 5:**
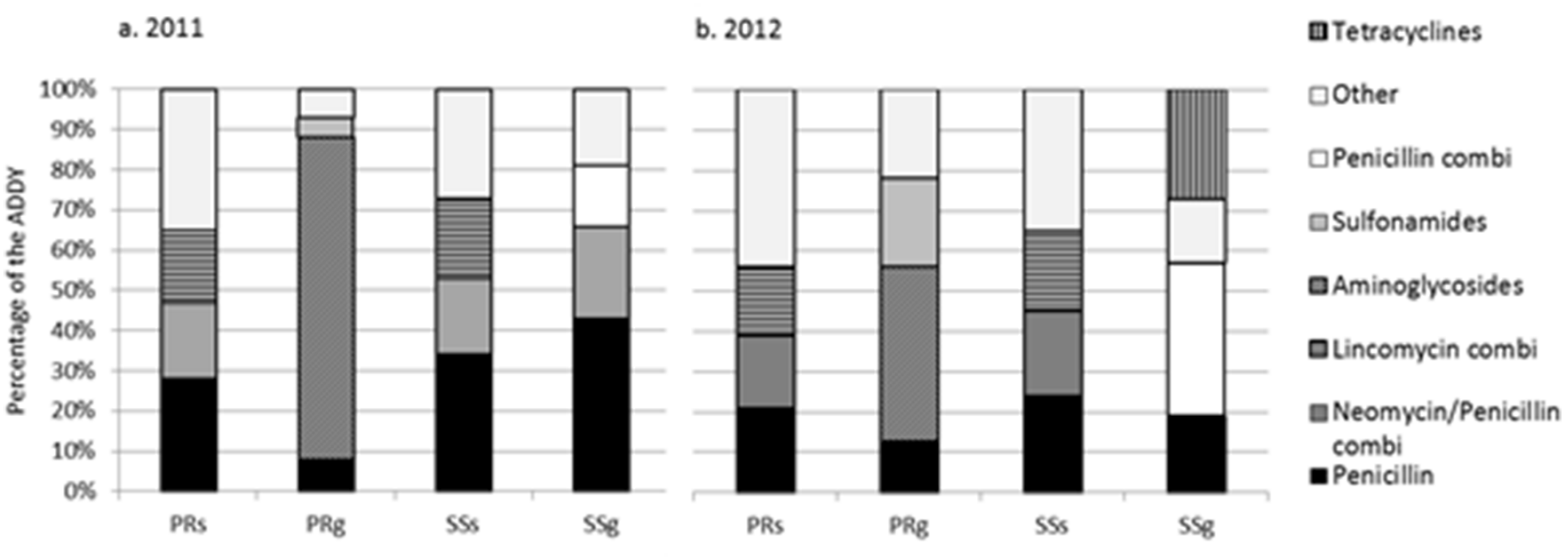
The distribution of the three most frequently used classes of active substances, and the rest summarized in the category “other”, presented as percentages of the total estimated animal used daily dose of antibiotics per year (AUDD/Y) in 2011 (a) and 2012 (b) per subtype of small ruminant farms in the Netherlands (PRs: professional sheep farms (≥32 sheep), PRg: professional goat farms (≥32 goats), SSs: small scale sheep farms (<32 sheep), SSg: small scale goat farms (<32 goats). The category “other” can, besides the 7 classes already described in the legend of the figure, also contain TMPS, Polymixins, Macrolides, Lincomycins, Fluoroquinolones, Fenicoles, Cephalosporins.

The AUDD/Y per subtype of farms with small ruminants was subdivided into 14 classes based on the active substance of the antibiotic. Penicillin was most commonly used, both in 2011 and in 2012, in small scale sheep and goat farms and in professional sheep farms (Figure 4). In the professional goat farms, antibiotics containing aminoglycosides were the most used class (Figure 5).

## Discussion

The median AUDD/Y in farms with small ruminants was 0 in both 2011 and 2012 (mean 0.62 and 0.60). The highest median values of the AUDD/Y were found in the professional goat industry. The small scale farmers had the lowest AUDD/Y values. In this study, all antibiotics that had been prescribed by one of the twelve veterinary practices to clients that kept at least one small ruminant were included. We decided to select veterinary practices rather than individual farmers, because in this way it was possible, within the framework of the study, to include a large number of herds with small ruminants (N = 5,399). The disadvantage of this decision was that the farms included might not be fully representative for the whole small ruminant population in the Netherlands. However, it appeared that parameters such as the ratio of herds between different subtypes, and herd sizes of farms included, were comparable to those of the entire small ruminant population. Therefore it was concluded that the results presented in this study give an accurate representation of antibiotic use in the entire small ruminant population in the Netherlands. Nevertheless, the AUDD/Y calculated in this study might be slightly biased because it was not possible to include farms that 1) did not have an UHI number or 2) did have an UHI number but were not registered in the I&R system. This was the case in 24% and 21% of the farms with antibiotic use in 2011 and 2012 respectively, and in 28% of the farms without antibiotic use. From January 1^st^ 2010 onwards, all farms with small ruminants are obliged to register their animals and animal movements in the I&R database. This database improves every year, but is not yet complete. After some additional research in cooperation with the veterinary practices, it was concluded that the majority of farms with missing I&R data were small herds that only kept a few small ruminants. The descriptive results of small ruminant farms to which antibiotics had been prescribed but for which no AUDD/Y could be calculated, showed that antibiotics were prescribed less frequently to these farms compared to farms with complete data (results not presented). Therefore it was concluded that removal of these farms from the analyses probably has resulted in a slight overestimation of the AUDD/Y in small ruminant farms.

Antibiotics were not prescribed to all farms with small ruminants in 2011 or 2012. According to the participating veterinarians this was as expected, because the likelihood that a farmer with only a few small ruminants would need antibiotics every year was relatively small, especially when these animals are only kept for companion purposes. Nevertheless, prescription of antibiotics to these small scale farms might lead to a theoretical overestimation of the AUDD/Y because veterinarians are obliged to supply a complete bottle of antibiotics while this bottle might not be used completely. This was also apparent in our data on the amount of antibiotic deliveries. If Small scale goat or sheep holders used antibiotics, then the AUDD/Y in their flock was higher than in the professional sheep farms and comparable to the AUDD/Y in professional goat farms.

We had access to the exact quantity of antibiotics that the participating veterinary practices delivered to small ruminant herds. It was unknown whether these antibiotics were actually used and if they were used at the prescribed dose. Nevertheless, for small ruminant herds in the Netherlands, antibiotics are almost exclusively prescribed to treat clinical signs, which makes it very likely that the prescribed antibiotics were used immediately after delivery. In addition, the possible overestimation in AUDD/Y we assume in small scale farms, is expected to be small in large scale farms. In these herds, in almost all cases complete bottles of antibiotics will be used and if antibiotics remain after treatment, these will be used on a later notice. Although we believe that the obligatory rule of delivering bottles instead of millilitres might have led to a slight overestimation in our calculations, this will only play a minor role because most antibiotics are used by large scale farms in which antibiotic delivery gives a reliable indication of the actual use.

It was remarkable that antibiotics were only prescribed to 47% and 58% of the professional sheep farms in 2011 and 2012 respectively. This percentage did not increase with increasing numbers of animals per farm (>150 sheep; 46% in 2011, and 60% in 2012). This was not as expected, because beforehand it was hypothesized that the large majority of these professional sheep farms would need to use antibiotics every year. This hypothesis was supported by the fact that a Canadian study found that 94% of the sheep herds used antibiotics during a year [16]. Nevertheless, the herds in their study were much larger (average flock size of 197 ewes) compared to the professional sheep farms in this study. The finding that for a part of the professional sheep farms no antibiotics were prescribed in one or both years was discussed with the veterinary practices. The veterinarians indicated that 1) a part of the farms were probably no longer affiliated to the veterinary practice concerned and 2) a part of the farms did receive antibiotics during the analysed years, but that these antibiotics were probably accidentally assigned to the cattle. Nevertheless, all veterinarians stated that they are becoming more and more aware of the importance of a correct registration of prescribed antibiotics, and declared that the registration in 2012 had already been improved compared to 2011. This was also visible in the data that showed an 11% increase in antibiotic use in professional sheep farms in 2012 compared to 2011. There were no indications that the quantity of antibiotics prescribed to herds with small ruminants had increased between 2011 and 2012, and therefore it was assumed that at least a part of this increase was caused by the improved quality of registration. Nevertheless, these flaws in registration might have led to a slight underestimation of the total AUDD/Y in the professional sheep farms.

In the small ruminant industry, a median AUDD/Y of 0 was found both in 2011 and in 2012. This was lower than the median AUDD/Y in veal (27.0 in 2011, and 21.0 in 2012), pigs (5.5 in 2011, and 6.2 in 2012), poultry (20.9 in 2011, and 17.1 in 2012) and cattle (1.5 in 2012) in the Netherlands [12]. The median AUDD/Y of small ruminants was comparable to the median AUDD/Y of suckling and fattening cows that also had a median AUDD/Y of 0. There are a few studies that looked at the number of antibiotic treatments in Canadian sheep, but in these studies the exact amount of prescribed and used antibiotics was unknown [16], [17]. To our knowledge, no studies have been published on the quantity of antibiotic use in small ruminants in other European countries, but as in the Dutch situation, antibiotic use in other livestock species appeared to be higher [18]–[21]. Although no information was available on antibiotic use in small ruminants in other European countries, we have no reasons to believe that there will be large differences, given the fact that the sheep and goat industry in these countries is more or less comparable to the Dutch situation.

Penicillin was most often used in sheep flocks and small scale goat herds, and aminoglycosides were mostly used in the professional goat herds. In Canadian sheep, antibiotics belonging to the penicillin class were also found to be most frequently used [16], [17]. In addition, tetracyclines were in the top three of most frequently used antibiotics in Canada, which is in accordance with our study.

In the entire livestock industry, aminoglycosides only play a minor role in the total quantity of prescribed antibiotics. Most antibiotics used in livestock in the Netherlands are tetracyclines or a combination of trimethoprim and sulfonamides. Products with penicillin, or a combination of penicillin and other active substances, were the third most used class of antibiotics in the Netherlands in 2012 [12]. In other countries, the most frequently used class of antibiotics also differs between different livestock species. However, in all species, antibiotics containing penicillin as the active substance were frequently used as well [19]–[22].

Because there is a relation between the use of antibiotics and development of antibiotic resistance [23], the use of antibiotics should be minimized as much as possible. The quantity of antibiotics used in the small ruminant industry appeared to be low with a median value of 0, which meant that more than 50% of the farms with small ruminants did not use any antibiotic during a whole year. This can partly be explained because a majority of farmers keep small ruminants on a small scale as companion animals. These farms only used antibiotics in sporadic cases. But even in the professional goat industry, median values of the AUDD/Y did not exceed the AUDD/Y of other livestock species that are kept in the Netherlands. Furthermore, in the Dutch small ruminant industry antibiotics appeared to be prescribed mostly to treat illness, while in other livestock industries these products were also used extensively on a prophylactic basis. Although the general use of antibiotics was found to be low, there appeared to be a small number of herds with small ruminants that had a very high AUDD/Y. It is recommended that these herds will be investigated more closely, to determine the reasons for the high rate of antibiotic use and to develop measures to reduce the use in these herds as appropriate.

This study aimed at quantifying the amount of antibiotics used in the small ruminant industry. We did not study the relation between the AUDD/Y and AMR. From a Canadian study it is known that in sheep, most AMR is found to tetracyclines [7]. However, these authors conclude that the overall AMR in Canadian sheep is low. Whether these results are also valid for small ruminants in the Netherlands is unknown and it is recommended to look into the relation of antibiotic use in Dutch small ruminants and AMR in more detail.

## Conclusion

In this study, the antibiotic use in the small ruminant industry in the Netherlands was estimated for the years 2011 and 2012. The median AUDD/Y was 0 both in 2011 and in 2012 (mean 0.62 and 0.60). This AUDD/Y is lower than this parameter reported in other livestock industries. Most antibiotics were prescribed to the professional goat industry. Nevertheless, the AUDD/Y in the professional goat industry was also lower compared to the AUDD/Y in other livestock industries. With the low usage of antibiotics in the small ruminant industry, it is likely that this industry might only play a minor role in the development of antibiotic resistance in the entire livestock industry.
